# Kinetic ^18^F-FDG PET/CT imaging of hepatocellular carcinoma: a dual input four-compartment model

**DOI:** 10.1186/s40658-024-00619-1

**Published:** 2024-02-22

**Authors:** Tao Wang, Yinglei Deng, Sidan Wang, Jianfeng He, Shaobo Wang

**Affiliations:** 1 Faculty of Information Engineering and Automation, Yunnan Key Laboratory of Artificial Intelligence, Kunming University of Science and Technology, Kunming, 650500 Yunnan China; 2 PET/CT Center, Affiliated Hospital of Kunming University of Science and Technology, First People’s Hospital of Yunnan, Kunming, 650031 China

**Keywords:** Hepatocellular carcinoma, PET/CT, Dual-input four-compartment, Kinetic model

## Abstract

**Background:**

The endoplasmic reticulum plays an important role in glucose metabolism and has not been explored in the kinetic estimation of hepatocellular carcinoma (HCC) via 18F-fluoro-2-deoxy-d-glucose PET/CT.

**Methods:**

A dual-input four-compartment (4C) model, regarding endoplasmic reticulum was preliminarily used for kinetic estimation to differentiate 28 tumours from background liver tissue from 24 patients with HCC. Moreover, parameter images of the 4C model were generated from one patient with negative findings on conventional metabolic PET/CT.

**Results:**

Compared to the dual-input three-compartment (3C) model, the 4C model has better fitting quality, a close transport rate constant (*K*_1_) and a dephosphorylation rate constant (*k*_6_/*k*_4_), and a different removal rate constant (*k*_2_) and phosphorylation rate constant (*k*_3_) in HCC and background liver tissue. The *K*_1_, *k*_2_, *k*_3_, and hepatic arterial perfusion index (*HPI*) from the 4C model and *k*_3_, *HPI*, and volume fraction of blood (*V*_*b*_) from the 3C model were significantly different between HCC and background liver tissues (all *P* < 0.05). Meanwhile, the 4C model yielded additional kinetic parameters for differentiating HCC. The diagnostic performance of the top ten genes from the most to least common was *HPI*(4C), *V*_*b*_(3C), *HPI*(3C), SUVmax, *k*_*5*_(4C), *k*_*3*_(3C), *k*_*2*_(4C), *v*(4C), *K*_*1*_(4C) and *V*_*b*_(4C). Moreover, a patient who showed negative findings on conventional metabolic PET/CT had positive parameter images in the 4C model.

**Conclusions:**

The 4C model with the endoplasmic reticulum performed better than the 3C model and produced additional useful parameters in kinetic estimation for differentiating HCC from background liver tissue.

**Supplementary Information:**

The online version contains supplementary material available at 10.1186/s40658-024-00619-1.

## Introduction

Primary liver cancer was the sixth most commonly diagnosed cancer and the third leading cause of cancer death worldwide in 2020, with hepatocellular carcinoma (HCC) accounting for 75–85% of cases [1].

18F-fluoro-2-deoxy-d-glucose (^18^F-FDG) positron emission tomography (PET)/computed tomography (CT) is a noninvasive imaging method for the diagnosis and evaluation of HCC that provides functional and molecular information [2, 3]. Dynamic PET/CT imaging enables accurate quantification of radiotracer inflow and uptake via kinetic analysis of ^18^F-FDG accumulation [4–7]. Continuous exploration has driven PET kinetics research, the advent of total-body PET has improved photon capture capabilities, and fast dynamic imaging with high temporal resolution has become feasible [5, 8, 9]. Moreover, more efficient algorithms are used to improve the accuracy of kinetic parameter estimation [10, 11].

A hypothetical kinetic model is critical and is proposed based on the biological process of drugs in cells to obtain kinetic parameters for the evaluation of physiological and pathological conditions to help cancer screening, diagnosis and treatment [12]. The two-compartment model merely describes tracer transport from blood to tissue, without further metabolic steps, and is appropriate only for transport markers. The three-compartment model describes the transport of markers between blood and tissue and the specific metabolic or binding target of radioactive markers [13, 14]. Wang et al. [15] proposed an optimized three-compartment model for the liver that adopted the optimally derived dual blood input function and the image-derived aortic input function and considered the dephosphorylation (*k*_4_) and fractional blood volume (*V*_*b*_) of ^18^F-FDG. The six parameters of the three-compartment model are generally accepted and consistent with those of liver ^18^F-FDG kinetic analysis [16–19].

The endoplasmic reticulum (ER) is the preferential site of ^18^F-FDG accumulation, and phosphorylated ^18^F-FDG enters the ER via the transmembrane protein glucose-6-phosphate transporter protein (G6PT), where it is hydrolysed to produce free ^18^F-FDG, which is further released into the cytoplasm [20, 21]. Cossu et al. [22] showed that glucose processing mechanisms in the ER contribute to brain FDG uptake. Scussolini et al. [23] used a four-compartment model with ER for kinetic analysis of mouse breast cancer cells and confirmed that ER plays a key role in ^18^F-FDG metabolism. Sommariva et al. [24] also showed that a four-compartment model with ER can also be used for ^18^F-FDG kinetic analysis in mouse colon cancer. However, the role of the four-compartment model for estimating liver ^18^F-FDG kinetics and distinguishing HCC remains unclear.

Liver kinetics require consideration of dual blood supplies from the hepatic artery and portal vein, and this study aimed to determine the following: (1) the availability of the dual-input four-compartment model with the endoplasmic reticulum (4C model) for liver and HCC kinetic analysis; (2) the differences in the corresponding kinetic parameters between the 4C model and the dual-input three-compartment (3C) model; (3) the role of the derived kinetic parameters for distinguishing HCC from background liver tissue; and (4) the HCC identifiability of the parametric images with the 4C model.

## Materials and methods

### Patients

The Institutional Review Board of the First People’s Hospital of Yun-nan Province approved this study (IRB number: KHLL2022- KY189). The 24 patients with HCC provided written informed consent to participate in the study; 21 males and 3 females were included. All patients underwent a 5-min dynamic PET/CT scan of the liver and a whole-body static PET/CT scan. A total of 28 pathologically diagnosed HCC lesions from 24 patients were analysed; 21 patients had a single HCC lesion, 2 patients had two HCC lesions, and 1 patient had three lesions. The long axis of these tumours ranged from 1.9 to 17.0 cm (mean 6.8 ± 3.5).

### PET/CT acquisition

All HCC patients were scanned using a Philips Ingenuity TF PET/CT scanner (Cleveland, OH, USA) after they had fasted for at least 6 h and relaxed in a quiet room with low ambient light.

### Dynamic PET/CT scan

A 5-min dynamic PET/CT scan was added before conventional PET/CT. A bolus injection was performed with ^18^F-FDG (5.5 MBq/kg) in 2 mL of 0.9% saline, which was subsequently flushed with 20 mL of 0.9% saline at a flow rate of 2 mL/s. A liver CT scan (120 kV, 100 mA) was performed in a single bed, and the liver was in the centre of the scanner’s field of view. For dynamic analysis, the images were divided into 16 frames: 5 s/frame for the first 1 min (12 × 5 s) and 60 s/frame thereafter (4 × 60 s).

### Conventional PET/CT scan

Conventional static scans were performed approximately 60 min after the ^18^F-FDG push, and a whole-body CT scan (120 kV; 200 mA) was performed, including from the vertex of the skull to the proximal thigh, followed by a 1-min PET scan in each bed and a total of 11 beds.

### Image analysis

The SUVmax was measured by delineating regions of interest (ROIs) on PET images following the methodology described in previous studies [11, 25]. Briefly, for lesions with imperceptible FDG uptake, ROIs were drawn relative to the conventional imaging findings. ROIs of the aorta and portal vein were placed at approximately two-thirds of the vascular cross-section. To compare HCC tumours to background tumour-free liver tissue, the respective ROIs were drawn in tumour-free liver tissue, and all the ROIs avoided blood vessels.

### Kinetic modelling

**Fig. 1:**
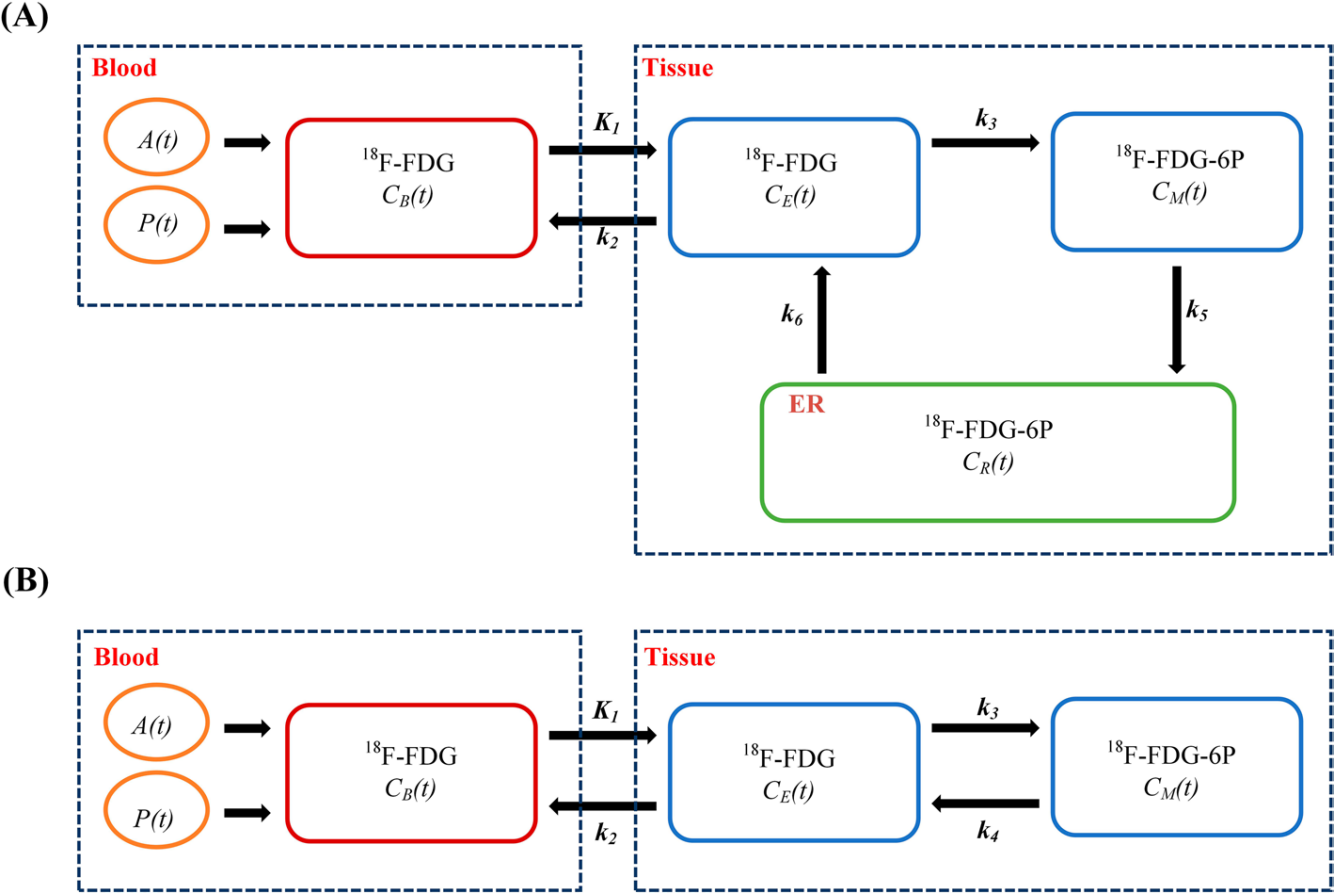
Compartmental model for liver kinetic modelling. **A** The 4C model. **B** The 3C model

In Fig. 1, the 4C model is compared to the 3C model. Figure 1A shows the 4C model, which accounted for the effect of the dual blood supply to the liver; the blood concentration of ^18^F-FDG ${\text{C}}_{{\text{B}}} \left( {\text{t}} \right)$, an input to the model, was calculated as the hepatic arterial ^18^F-FDG concentration *A(t)* and portal venous ^18^F-FDG concentration *P(t)*:

1 $$\begin{array}{*{20}c} {C_{B} \left( t \right) = HPI \times A\left( t \right) + \left( {1 - HPI} \right) \times P\left( {\text{t}} \right)} \\ \end{array}$$

where *HPI* represents the hepatic artery perfusion index. ${\text{C}}_{{\text{E}}} \left( {\text{t}} \right)$ represents the concentration of ^18^F-FDG in liver tissue. ${\text{C}}_{{\text{M}}} \left( {\text{t}} \right)$ represents the concentration of phosphorylated ^18^F-FDG-6-phosphate (^18^F-FDG6P) in liver tissue. ${\text{C}}_{{\text{R}}} \left( {\text{t}} \right)$ represents the concentration of ^18^F-FDG6P in the ER. *K*_*1*_ is the rate constant for the transport of ^18^F-FDG from blood to tissue by GLUT, and *k*_*2*_ is the rate constant for removal. *k*_*3*_ is the rate constant for the phosphorylation of ^18^F-FDG to ^18^F-FDG6P, *k*_*5*_ is the input rate of ^18^F-FDG6P into the ER (by G6PT), and *k*_*6*_ is the dephosphorylation rate of ^18^F-FDG6P to ^18^F-FDG (by G6Pase).

The compartment model is equivalently described by a set of ordinary differential equations [16]:

2 $$\begin{array}{*{20}c} {\frac{dc\left( t \right)}{{dt}}c = MC\left( t \right) + K_{1} C_{B} \left( t \right)e, C\left( 0 \right) = 0} \\ \end{array}$$

The compact form of the IPE is given by:

3 $$\begin{array}{*{20}c} {C_{T} \left( t \right) = \alpha c\left( t \right) + V_{b} C_{B} \left( t \right)} \\ \end{array}$$

$C_{T} \left( t \right)$ is the output function of the kinetic model, which is the curve of tracer concentration in the tissue as measured by PET/CT images over time.

For the 4C model (Fig. 1A), we have:

4 $$\begin{array}{*{20}c} {M = \left( {\begin{array}{*{20}c} { - \left( {k_{2} + k_{3} } \right)} & 0 & {k_{6} } \\ {k_{3} } & { - k_{5} } & 0 \\ 0 & {k_{5} } & { - k_{6} } \\ \end{array} } \right), C\left( t \right) = \left( {\begin{array}{*{20}c} {C_{E} \left( t \right)} \\ {C_{M} \left( t \right)} \\ {C_{R} \left( t \right)} \\ \end{array} } \right), e = \left( {\begin{array}{*{20}c} 1 \\ 0 \\ 0 \\ \end{array} } \right), \alpha = \left[ {\begin{array}{*{20}c} {\alpha_{1} } \\ {\alpha_{2} } \\ {\alpha_{3} } \\ \end{array} } \right]} \\ \end{array}$$

where

5 $$\begin{array}{*{20}c} {\alpha_{1} = V_{i} + \left( {1 - v_{r} } \right)\left( {1 - V_{b} - V_{i} } \right)} \\ \end{array}$$

6 $$\begin{array}{*{20}c} {\alpha_{2} = \left( {1 - v_{r} } \right)\left( {1 - V_{b} - V_{i} } \right)} \\ \end{array}$$

7 $$\begin{array}{*{20}c} {\alpha_{3} = v_{r} \left( {1 - V_{b} - V_{i} } \right)} \\ \end{array}$$

8 $$\begin{array}{*{20}c} {v_{r} = \frac{v}{1 + v}} \\ \end{array}$$

$V_{b}$ and $V_{i}$ represent the blood and interstitial volume fractions, respectively, and $v$ represents the ratio between the ER and cytosolic volume.

For the 3C model (Fig. 1B), we have:

9 $$\begin{array}{*{20}c} {M = \left( {\begin{array}{*{20}c} { - \left( {k_{2} + k_{3} } \right)} & {k_{4} } \\ {k_{3} } & { - k_{4} } \\ \end{array} } \right), C = \left( {\begin{array}{*{20}c} {C_{E} \left( t \right)} \\ {C_{M} \left( t \right)} \\ \end{array} } \right), e = \left( {\begin{array}{*{20}c} 1 \\ 0 \\ \end{array} } \right),\alpha = \left[ {\begin{array}{*{20}c} {1 - V_{b} } \\ {1 - V_{b} } \\ \end{array} } \right]} \\ \end{array}$$

Notably, the *k*_*6*_ of the 4C model is comparable to the *k*_*4*_ of the 3C model.

### Parameter images

The aorta and portal vein were drawn as ROIs for an input function to the 4C model, and the formulas for calculating the parameters were as described above. Constrained by the fitting algorithm and kinetic model, we selected specific parameters of a patient whose lesion was detected via negative PET/CT images for pixel-by-pixel kinetic modelling. *K*_*1*_, *k*_*3*_, *k*_*5*_ and *HPI* images were generated by performing 17 sequential frames of dynamic PET on a 144 × 144-pixel matrix image for each HCC patient. Pseudocolour was used for parameter images to display the calculated value in each pixel. The pseudocolour values for *K*_1_, *k*_3_ and *HPI* were greater for HCC lesions than for background liver tissue and lower for *k*_5_. CT imaging was used to determine the exact location of the lesion.

### Parameter estimation

With the rate constants as fit parameters, all model fits were performed according to the least-squares method, optimized with the Levenberg–Marquardt algorithm and implemented using MATLAB, R2019a (MathWorks, Natick, MA, USA). The unknown model parameter set of the 4C model is *θ* = $\left[ {K_{1} ,k_{2} ,{ }k_{3} ,{ }k_{5} ,{ }k_{6} ,HPI,V_{b} ,V_{i} , v} \right]$, and that of the 3C model is *θ* = $\left[ {K_{1} ,k_{2} ,{ }k_{3} ,{ }k_{4} ,{ }HPI,{ }V_{b} } \right]$:

10 $$\begin{array}{*{20}c} {\hat{\theta } = \mathop {{\text{argmin}}}\limits_{\theta } WRSS\left( \theta \right)} \\ \end{array}$$

11 $$\begin{array}{*{20}c} {WRSS\left( \theta \right) = \mathop \sum \limits_{i = 1}^{N} w_{i} \left[ {c_{i} - C_{T} \left( {t_{i} ;\theta } \right)} \right]} \\ \end{array}$$

where *WRSS(θ)* represents the weighted residual sum of squares of the curve fit and $w_{i}$ represents the weighting factor of time frame *N*.

### Statistical analysis

The TAC fit quality between the 4C model and the 3C model was compared using the Akaike information criterion (AIC) [26, 27]. Statistical analysis was performed using MedCalc version 13.0.0.0 (MedCalc software, Ostend, Belgium). Receiver operating characteristic (ROC) analysis was used to compare the kinetic parameters between HCC tissues and background liver tissues. The *P* values were calculated based on paired Student’s *t* tests, and *P* < 0.05 indicated significant differences.

## Results

### TAC fit quality

**Fig. 2:**
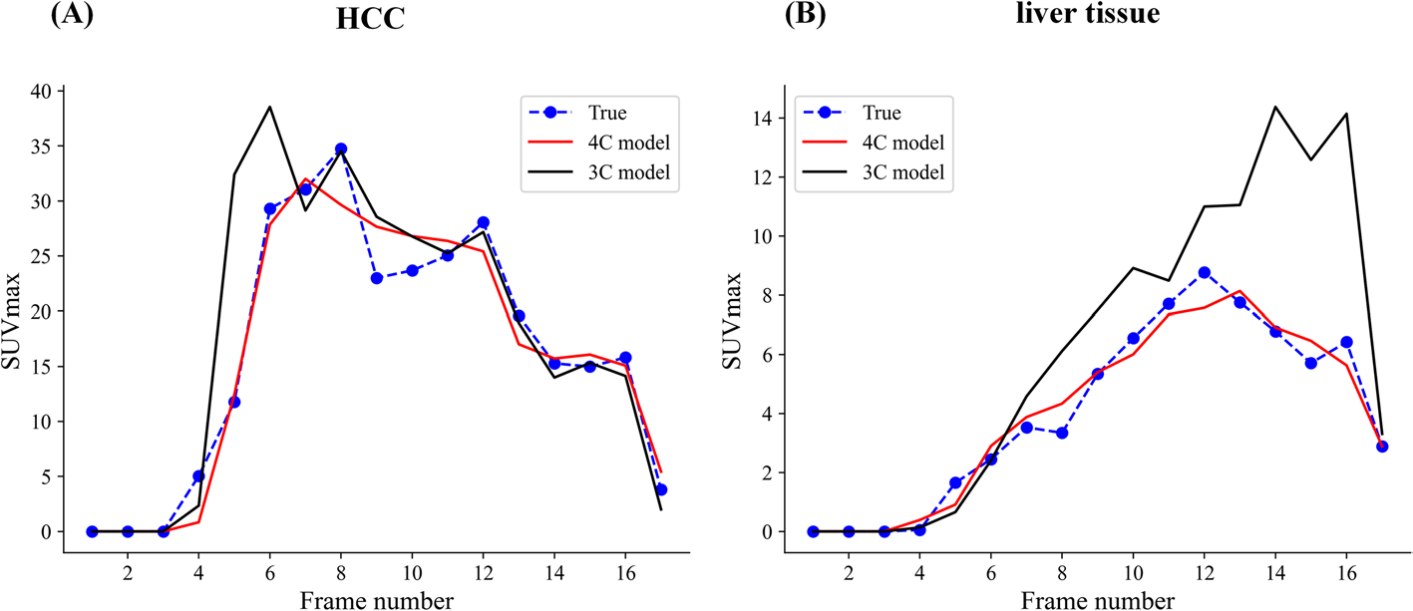
Examples of TAC fitting for two models. **A** TAC fitting for background liver tissue. **B** TAC fitting for HCC

The 4C model had a better fit quality with lower AIC values than did the 3C model for most patients, as shown in Table 1 and Additional file 1: Figure S1. An example of TAC fitting results for a patient with background liver tissue and HCC is shown in Fig. 2, with the 4C model fitting the data better than the 3C model.

**Table 1:** The AIC of the two models

Model	HCCs	Liver tissues
	n = 28	n = 24
Dua-input four-compartment model	80.92 ± 29.43	52.20 ± 32.78
Dua-input three-compartment model	86.05 ± 16.28	58.74 ± 25.01
*P*	0.286	0.025

### Differences in the corresponding kinetic parameters between the 4C and 3C models

The differences in the corresponding kinetic parameters between the 4C and 3C models were compared, as shown in Table 2.

**Table 2:** Differences in the corresponding kinetic parameters between the 4C model and 3C model

	HCC			Liver tissue		
	4C model	3C model	*P*	4C model	3C model	*P*
*K*_1_	1.441 ± 0.314	1.302 ± 0.294	0.210	1.179 ± 0.465	1.235 ± 0.418	0.515
*k*_2_	1.018 ± 0.448	1.435 ± 0.298	**< 0.001**	0.704 ± 0.491	1.260 ± 0.406	**< 0.001**
*k*_3_	1.348 ± 0.216	0.047 ± 0.055	**< 0.001**	1.103 ± 0.509	0.017 ± 0.030	**< 0.001**
*k*_6_*/k*_4_	0.037 ± 0.050	0.026 ± 0.040	0.259	0.038 ± 0.053	0.041 ± 0.053	0.923

Bold values indicate significant differences in this parameter

The two models were close for *K*_1_ (*P* = 0.210, *P* = 0.515) and *k*_6_/*k*_4_ (*P* = 0.259, *P* = 0.923) but different for *k*_2_ (*P* < 0.001, *P* < 0.001) and *k*_3_ (*P* < 0.001, *P* < 0.001) in HCC and background liver tissue.

### Comparison of kinetic parameters for distinguishing HCC from background liver tissue between the 4C and 3C models

The parameters obtained from the kinetic modelling analysis of liver ^18^F-FDG PET/CT data using the 4C model and 3C model are shown in Table 3.

**Table 3:** Kinetic parameters estimated with the 4C model and 3C model

Model	Parameters	HCC	Liver tissue	AUC	*P*
4C	*K*_1_	1.441 ± 0.314	1.179 ± 0.465	0.636	**0.019**
	*k*_2_	1.018 ± 0.448	0.704 ± 0.491	0.698	**0.020**
	*k*_3_	1.348 ± 0.216	1.103 ± 0.509	0.629	**0.022**
	*k*_5_	0.978 ± 0.490	1.372 ± 0.506	0.734	**0.006**
	*k*_6_	0.037 ± 0.050	0.038 ± 0.053	0.599	0.956
	*HPI* (%)	0.836 ± 0.237	0.254 ± 0.286	0.916	**< 0.001**
	*V*_*b*_	0.075 ± 0.133	0.029 ± 0.058	0.636	0.127
	*Vi*	0.540 ± 0.399	0.286 ± 0.430	0.622	**0.032**
	*v*	0.317 ± 0.438	0.063 ± 0.215	0.668	**0.013**
	All*			0.931	
3C	*K*_1_	1.302 ± 0.294	1.235 ± 0.418	0.541	0.498
	*k*_2_	1.435 ± 0.298	1.260 ± 0.406	0.607	0.080
	*k*_3_	0.047 ± 0.055	0.017 ± 0.030	0.719	**0.021**
	*k*_4_	0.026 ± 0.040	0.041 ± 0.053	0.552	0.239
	*HPI* (%)	0.652 ± 0.328	0.207 ± 0.211	0.864	**< 0.001**
	*V*_*b*_	0.208 ± 0.176	0.043 ± 0.062	0.875	**< 0.001**
	All*			0.892	
SUVmax		5.131 ± 2.776	2.791 ± 0.711	0.814	**< 0.001**

Bold values indicate significant differences in this parameter

*The multiparametric ROC

*K*_1_, *k*_2_, *k*_3_ and *HPI* were greater in HCC tissue than in background liver tissue (*P* = 0.019, *P* = 0.020, *P* = 0.022, *P* < 0.001) according to the 4C model, and *k*_6_ and *V*_b_ were not significantly different between diagnostic HCC and background liver tissue (*P* = 0.956, *P* = 0.127). Notably, *k*_5_ from the 4C model was lower in HCC tissue than in background liver tissue (*P* = 0.006). *V*_*i*_ and *v* were greater in HCC tissue than in background liver tissue (*P* = 0.032, *P* = 0.013).

*k*_3_, *HPI* and *V*_*b*_ according to the 3C model were greater in HCC tissue than in background liver tissue (*P* = 0.021, *P* < 0.001,* P* < 0.001), and *K*_1_, *k*_2_ and *k*_4_ were not significantly different between HCC tissue and background liver tissue (*P* = 0.498, *P* = 0.080,* P* = 0.239).

The diagnostic performance of the top ten from high to low is *HPI*(4C), *V*_*b*_(3C), *HPI*(3C), SUVmax, *k*_5_(4C), *k*_3_(3C), *k*_2_(4C), *v*(4C), *K*_1_(4C) and *V*_*b*_(4C).

A multiparametric ROC curve was used to compare the diagnostic performance of all the parameters of the 4C model and the 3C model. The AUC values of the multiparametric ROC curves of the 4C model and 3C model were 0.931 and 0.892, respectively.

### Parameter images

**Fig. 3:**
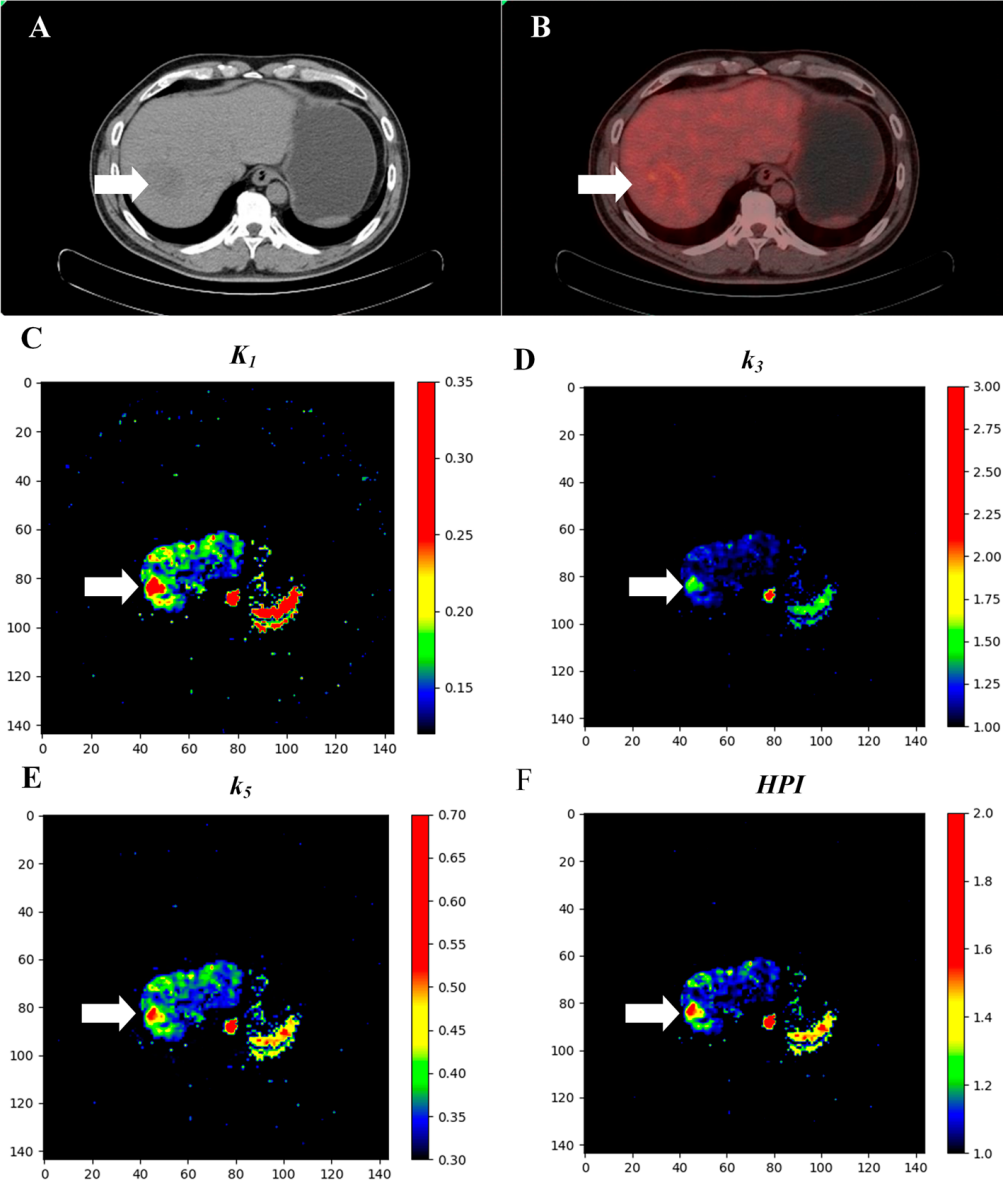
The parametric images from the 4C model show hepatocellular carcinoma (arrow) with negative findings on conventional metabolic PET/CT. **A** CT image. **B** Conventional static PET/CT image. **C** Parameter image of *K*_1_. **D** Parameter image of *k*_*3*_. **E** Parameter image of *k*_5_. **F** Parameter image of *HPI*

The parameter images of* K*_1_, *k*_3_, *k*_5_ and *HPI* of the 4C model were generated using the pixel-by-pixel method. Figure 3 shows a comparison of images from a 72-year-old male patient. CT image showing a slight decrease in opacity in the left lobe of the liver. Conventional static PET/CT images revealed negative results in the liver.

In the 4C model, the parameter images for *K*_1_ (Fig. 3C), *k*_3_ (Fig. 3D), *k*_5_ (Fig. 3E) and *HPI* (Fig. 3F) identified HCC (marked with arrows) in a patient who had negative findings on conventional metabolic PET/CT.

## Discussion

This study preliminarily demonstrated the feasibility of the 4C model for short-term dynamic PET/CT in HCC patients. Compared with previous studies in which a 3C model was used to estimate ^18^F-FDG metabolism in the liver [28, 29], the 4C model has more kinetic parameters that can distinguish HCC from background liver tissue, and it provides additional kinetic parameters with diagnostic efficacy and, in addition, preliminarily generated parameter images to diagnose HCC.

Compared to static PET/CT imaging, dynamic PET/CT imaging with a kinetic model has the potential for use in all multiparameter quantitative imaging and more accurate metabolic information, and it is widely used for diagnosis [17, 30, 31]. Dynamic PET protocols are less suitable for routine clinical application because of the longer scanning times, which can be too great of a burden to the patient. Liu et al. [5] performed kinetic modelling for different tissues or organs at 30, 45, and 75 min. Samimi et al. [32] proposed a short-term PET protocol using an early 5-min dynamic, supplemented by 3-min static imaging at 60 min postinjection. Based on the findings of previous studies and our clinical practice [11, 25, 33], a short-term protocol was used in this study, with 5-min dynamic PET supplemented by 1-min static imaging at 60 min postinjection; because of the metabolism of ^18^F-FDG, static images were considered.

The three-compartment model describes the transport and phosphorylation of ^18^F-FDG and is promising for differential diagnosis and therapeutic assessment of HCC. The development of cancer has further complicated the quantitative analysis of ^18^F-FDG. G6P-phosphatase (G6Pase) is a complex of multiple proteins anchored to the ER lumen whose expression has been mostly characterized in the liver, kidney and gut; FDG6P is a recognized substrate for this enzyme, and its activity explains the relatively low sensitivity of ^18^F-FDG PET/CT in hepatocellular carcinoma [34, 35]. The ER plays a crucial role in the activation of G6Pase, and ^18^F-FDG6P and GP6 are transported via the transmembrane protein glucose-6-phosphate transporter (G6PT) into the ER, where hydrolysis produces free ^18^F-FDG molecules that are released into the cytoplasm [22].

The experimental results of this study showed that the two models could fit the TAC well, and the 4C model showed better fit quality due to the greater number of parameters. The overall diagnostic efficacy of the 4C model was greater than that of the 3C model according to multivariate ROC analysis.

The liver has unique physiological features, as it has a dual blood supply from the hepatic artery and portal vein. HCC is supplied with most of its blood flow by the hepatic artery, which accounts for approximately 75–80%, whereas only 20–25% of normal liver tissue is supplied. Geist et al. [28] assessed the three-compartment model with different input functions, and the results showed that the kinetic parameters of the liver can be better estimated with a dual-input function. Wang et al. [18] used the SUVmean and SUVmax to model liver kinetics, and the proportion of hepatic arterial supply was greater in both HCCs than in normal liver tissue. We derive the portal vein input function directly from the image instead of from the spleen. First, ^18^F-FDG exhibits a heterogeneous distribution in the arterial phase of the spleen. Second, the spleen contains both blood pool ^18^F-FDG and metabolized ^18^F-FDG. The results of the present study were consistent with those of previous studies in that the *HPI* was significantly increased in HCC patients in the 3C and 4C models, and the *HPI* in the 4C model was closer to the theoretical clinical value (0.836 ± 0.237 vs. 0.254 ± 0.286).

This study compared 4C and 3C models, which have similar biological significance and similar pharmacokinetic process parameters. The experimental results showed that the two models had similar transport and dephosphorylation rate constants and different removal and phosphorylation rate constants.

*K*_*1*_ is the rate of transport of ^18^F-FDG from blood to tissue and is of great clinical importance. Wang et al. [15] showed that* K*_1_ was able to assess liver inflammation in a staged manner. Zuo et al. [36] also showed the potential value of *K*_1_ in assessing liver inflammation. The results of this study showed that *K*_1_ expression is greater in HCC tissue than in background liver tissue in both models, which is due to the high expression of glucose transporter proteins in tumour cells. However, the 3C model of *K*_1_ has no diagnostic efficacy. There was no significant difference in the *K*_1_ values between the 4C model and the 3C model. Sommariva et al. [24] also reported similar estimates of *K*_1_ for both models in a kinetic model of mouse colon cancer cells.

*k*_2_, a marker of clearing ^18^F-FDG in tissue, was greater in HCC tissue than in background liver tissue in both models, and the 4C model showed significant differences. Notably, *k*_2_ was not consistent between the two models, and that of the 4C model was lower than that of the 3C model for HCC and background liver tissue. The 4C model assumes that the endoplasmic reticulum is involved in the metabolism of ^18^F-FDG and that the amount of free ^18^F-FDG in the cytoplasm is reduced.

When ^18^F-FDG enters tissues, hexokinase is phosphorylated to form ^18^F-FDG-6P. Hexokinase is highly expressed in HCC, and *k*_3_ is the phosphorylation rate. The results of this study showed that the *k*_3_ values of the two models could both be used to distinguish HCC tissue from background liver tissue. However, their values were not consistent, with the *k*_3_ of the 3C model being lower than that of the 4C model. Cossu et al. [22] demonstrated that ER metabolism may lead to further degradation of ^18^F-FDG-6P. In the 4C model, ^18^F-FDG-6P enters the endoplasmic reticulum through the transmembrane protein glucose-6-phosphate translocase, disrupting metabolic homeostasis in the cytoplasm, enhancing the catalytic activity of hexokinase, and producing more ^18^F-FDG-6P to maintain a dynamic balance in the cell.

Glucose-6-phosphatase plays a critical role in maintaining blood glucose homeostasis, and it is highly expressed in the liver, which leads to the dephosphorylation of ^18^F-FDG-6P. In contrast to that in the 3C model, ^18^F-FDG-6P in the 4C model enters the ER (*k*_5_), where it undergoes dephosphorylation and generates free ^18^F-FDG, which is released into the cytoplasm. The results of this study showed that *k*_5_ could be used to distinguish HCC tissue from background liver tissue, and background liver tissue values of *k*_5_ were significantly greater than those in HCC tissue, indicating low expression of glucose-6-phosphatase in HCC. *k*_6_ in the 4C model and *k*_4_ in the 3C model represent the rate of dephosphorylation of ^18^F-FDG-6P, and their values are basically the same, which is consistent with the results of previous studies [22, 24]. The rate of dephosphorylation is very low, but neglecting this parameter results in an underestimation of the ^18^F-FDG metabolic rate.

The blood volume parameter *V*_*b*_ can be measured using imaging methods [37]; however, this increases the complexity of PET imaging protocols and the radiation dose of ^18^F-FDG. The results of this study showed that the *V*_*b*_ of HCC tissue was greater than that of background liver tissue and were smaller with the 4C model. The 4C model includes extra compartments to account for more complex dynamics, such as differentiating between cellular and extracellular spaces or accounting for specific receptor-binding sites. These additional compartments may affect the estimation of Vb, making Vb appear smaller in the 4C model because the tracer distribution is accounted for in more compartments than in the 3C model.

Notably, in addition to *k*_5_, the 4C model also derives other kinetic parameters, namely, the volume fraction of interstitial *V*_*i*_ and the ratio of ER to cytosolic volume *v*. The measurement of these two parameters is complicated, and they are taken as the estimation parameters in this paper. The experimental results indicate that, compared with background liver tissue, *V*_*i*_ and *v* exhibit higher levels in HCC tissues. These findings could be used to distinguish HCC tissues from background liver tissue. These findings may be closely related to tumour growth and provide a theoretical basis for exploring the process of glucose uptake.

Due to the limitations of the fitting algorithm and model complexity, pixel-by-pixel calculations with multiple parameters are not applicable, and pixel-by-pixel dynamic modelling with specific kinetic parameters can better evaluate whole-liver uptake. Compared with other kinetic parameters, *K*_1_, *k*_3_, *k*_5_ and *HPI* have more important clinical value. Therefore, we further performed pixel-by-pixel kinetic modelling in a patient with negative findings on conventional metabolic PET/CT to generate parametric images of four parameters using a 4C. Our results showed that the use of parametric images is helpful for visualizing quantitative parameters of whole-liver tracer kinetics and adds a new dimension to the existing conventional PET or PET/CT images.

The present study has several limitations. First, the sample size of the dataset was small. Second, the reconstruction algorithm may affect the SUV [38]; however, further research into the use of the reconstruction algorithm is needed to improve the image quality of dynamic PET. Third, the pixel-by-pixel method takes a long time to generate parametric images, and further studies are needed to improve the fitting algorithm to increase the computational speed. Finally, due to practical and ethical considerations, kinetic analysis studies of lesion staging and drug treatments in human subjects are limited, and this topic will be the direction of future clinical tracer kinetics research.

## Conclusion

In this study, we propose a 4C model with an endoplasmic reticulum for liver kinetics modelling via dynamic ^18^F-FDG PET/CT imaging. The results showed that the 4C model performed better than the 3C model and produced additional useful parameters for kinetic estimation for differentiating HCC from background liver tissue, and the derived parameter images might be useful for diagnosing HCC.

### Supplementary Information

**Additional file 1**. **Figure S1**. Comparison of fitting quality for each.

## Data Availability

The datasets generated and/or analysed during the current study are available from the corresponding author upon reasonable request.
