# Supplementary figures and images for: Kinetic 18F-FDG PET/CT imaging of hepatocellular carcinoma: a dual input four-compartment model
Source: EJNMMI Phys. 2024 Feb 22;11:20. doi: 10.1186/s40658-024-00619-1 (PMC10884391; doi:10.1186/s40658-024-00619-1)

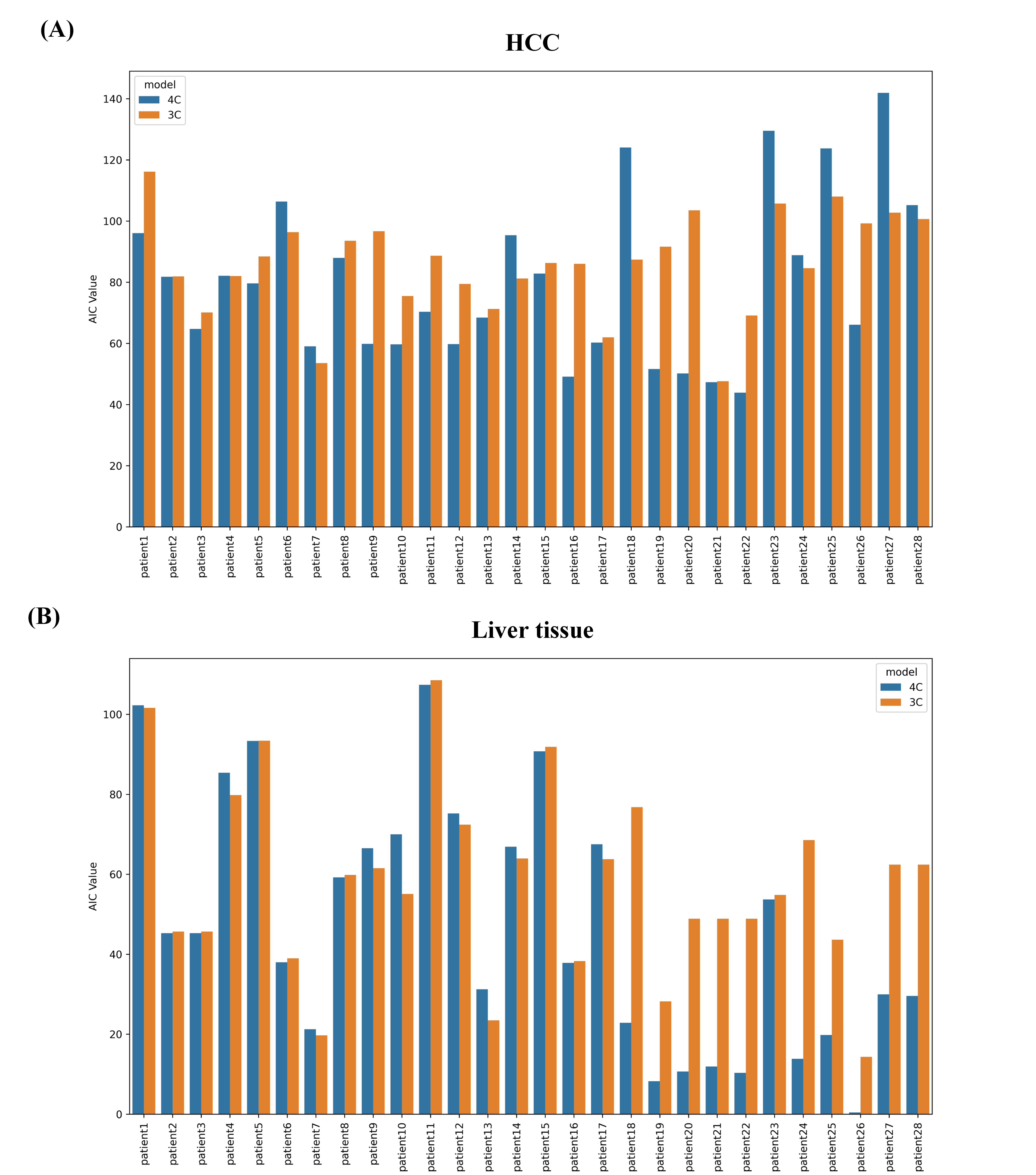

Supplement: Supplementary file 1 — Additional file 1. Figure S1. Comparison of fitting quality for each. [file 40658_2024_619_MOESM1_ESM.png]
